# Malglycemia is strongly associated with increased risk of ICU-acquired infection

**DOI:** 10.1186/cc9818

**Published:** 2011-03-11

**Authors:** J Krinsley, MJ Schultz, TS Hall, S Krasnica, JC Preiser

**Affiliations:** 1 Stamford Hospital, Stamford, CT, USA; 2 Academic Medical Center, Amsterdam, the Netherlands; 3 Erasme University Hospital, Brussels, Belgium

## Introduction

Infections that develop after admission to the ICU cause substantial morbidity and increases in resource utilization. The purpose of this investigation was to study the relationship of malglycemia, defined as blood glucose level (BGL) <70 or >139 mg/dl and the risk of developing ICU-acquired infection, occurring more than 2 days after ICU admission.

## Methods

This is a retrospective evaluation of prospectively collected data from the ICU's clinical database. Infection control nurses using standard definitions prospectively identified ICU-acquired infections.

## Results

A total of 3,263 patients were admitted to a medical-surgical ICU between 1 December 2007 and 31 May 2010 and had at least three BGL measurements. In this group, 142 (4.4%) patients developed 171 infections. Patients who developed infection had significantly longer ICU length of stay (*P *< 0.0001), higher scores for severity of illness (mean APACHE IV predicted mortality 34.8% vs. 18.5%, *P *< 0.0001) and greater mortality (32.4% vs. 13.3%, *P *< 0.0001) than did those who did not develop infection. Of 505 patients who did not have a single episode of malglycemia, none developed infection. In contrast, 16.8%, 13.1% and 7.2% of patients with lowest BGL <40, 40 to 54 and 55 to 69 mg/dl as well as 0.8%, 3.3%, 5.7% and 9.6% of patients with highest BGL 140 to 159, 160 to 179, 180 to 249 and >249 mg/dl developed infection. Multivariable logistic regression analysis identified BGL <70 and >139 mg/dl as independent predictors of the risk of developing infection (odds ratio (95% CI) 2.23 (1.45 to 3.44), *P *= 0.003 and 13.94 (1.91 to 101.90), *P *= 0.0094, respectively).

## Conclusions

Malglycemia is strongly associated with increased risk of ICU-acquired infections. Efforts to decrease the rate of hypoglycemia and hyperglycemia may reduce morbidity in the ICU.

